# Differential physiological and metabolic response to low temperature in two zoysiagrass genotypes native to high and low latitude

**DOI:** 10.1371/journal.pone.0198885

**Published:** 2018-06-11

**Authors:** Shuangming Li, Yong Yang, Qiang Zhang, Ningfang Liu, Qingguo Xu, Longxing Hu

**Affiliations:** 1 Department of Pratacultural Sciences, College of Agronomy, Hunan Agricultural University, Changsha, Hunan, China; 2 Golf College, Hunan International Economics University, Changsha, Hunan, China; Hainan University, CHINA

## Abstract

Low temperature is one of the important limiting factors for growing season and geographical distribution of plants. Zoysiagrass (Zoysia Willd) is one of the widely used warm-season turfgrass that is distribute in many parts of the world. Zoysaigrass native to high latitude may have evolved higher cold tolerance than the ones native to low latitude. The objective of this study was to investigate the cold stress response in zoysiagrass native to diverse latitude at phenotypic, physiological and metabolic levels. Two zoysiagrass (*Z*. *japonica*) genotypes, Latitude-40 (higher latitude) and Latitude-22 (lower latitude) were subjected to four temperature treatments (optimum, 30/25°C, day/night; suboptimum, 18/12°C; chilling, 8/2°C; freezing, 2/-4°C) progressively in growth chambers. Low temperature (chilling and freezing) increased leaf electrolyte leakage (EL) and reduced plant growth, turf quality, chlorophyll (Chl) content, photochemical efficiency (Fv/Fm) and photosynthesis (P_n_, net photosynthetic rate; g_s_, stomatal conductance; intercellular CO_2_; T_r_, transpiration rate) in two genotypes, with more rapid changes in Latitude-22. Leaf carbohydrates content (glucose, fructose, sucrose, trehalose, fructan, starch) increased with the decreasing of temperature, to a great extend in Latitude-40. Leaf abscisic acid (ABA), salicylic acid (SA) and jasmonic acid (JA) content increased, while indole-3-acetic acid (IAA), gibberellic acid (GA_3_) and trans-zeatin ribside (t-ZR) content decreased with the reduction of temperature, with higher content in Latitude-40 than in Latitude-22. Chilling and freezing induced the up-regulation of C-repeat binding factor (*ZjCBF*), late embryogenesis abundant (*ZjLEA3*) and dehydration-responsive element binding (*ZjDREB1*) transcription factors in two genotypes, whereas those genes exhibited higher expression levels in Latitude-40, particularly under freezing temperature. These results suggested that zoysiagrass native to higher latitude exhibited higher freezing tolerance may attribute to the higher carbohydrates serving as energy reserves and stress protectants that stabilize cellular membranes. The phytohormones may serve signals in regulating plant growth, development and adaptation to low temperature as well as inducing the up-regulated *ZjCBF*, *ZjLEA3* and *ZjDREB*1 expressions thus result in a higher cold tolerance.

## Introduction

Low temperature is the primary determining factor limiting geographical distribution and growing season of plants [1]. The injury symptoms are often exhibited at temperatures below 12°C for plant species native to tropical and subtropical areas [2]. However, the injuries caused by low temperature is generally categorized into chilling stress (temperatures above 0°C) and freezing stress (temperatures below 0°C) [3]. Chilling stress is often leading to growth and photosynthesis reduction, leaf wilting and chlorosis or even necrosis, cellular membrane damages as well as oxidative stress in plants [4]. In addition, temperatures declined below 0°C caused freezing stress, which result in the formation of ice crystals within the cell, mechanical damages as well as metabolic dysfunction in plants [5]. However, plants have evolved complex mechanisms to tolerate chilling and freezing stresses, such as accumulation of carbohydrates and proteins, resulting in large quantities of soluble sugars, amino acids and cold induced stress-related proteins [6], as well as hormone homeostasis inducing the gene expression that function to stabilize membranes against freezing-induced injury [7–8].

The acquisition of freezing tolerance called cold acclimation, which is generally initiated after a period of decreases in above 0°C temperatures and photoperiod [9]. The growth inhibition resulting from low temperature reduced the capacity for energy utilization that lead to feedback inhibition of photosynthesis [3]. Differences in the capacity to minimize photoinhibition and recover photosynthesis during cold acclimation have been shown to contribute to intra- and inter-specific differences in freezing tolerance [10]. Carbohydrates are synthesized from a complex series of reactions involving photosynthesis in plants [3]. Changes in the three major types of water-soluble carbohydrates monosaccharides (glucose and fructose), disaccharides (sucrose, trehalose) and fructans have often been reported in plants subjected to low temperature [11]. Those sugars may served as a typical osmoprotectant, cellular membrane stabilizer, scavengers of reactive oxygen species and signaling molecules in plants to tolerant chilling and freezing stress [11]. Many studies have showed that higher accumulation of sugars, or individual sugar fractions such as starch [12], fructans [13] and sucrose [12] during cold acclimation are associated with great cold tolerance in turfgrass [14–15]. Sucrose consists of the monosaccharides glucose and fructose, and its intimate involvement in growth, development, storage, signaling and stress acclimation [16]. Leaf starch potentially contributed carbon to carbohydrate accumulation in response to low temperature [17]. Fructans often accumulated during cold acclimation in cereals and grasses, which serve to stabilize cell membranes [18]. Furthermore, trehalose, a non-reducing disaccharide of glucose, has been implicated in modulating levels of freezing tolerance and may also be involved in starch accumulation [19].

Plant hormones may serve as signals in regulating plant growth, development and adaptation to environmental stress [20]. For abscisic acid (ABA), they can affect the diverse processes such as leaf senescence, seed dormancy and germination as well as cell division and elongation [21]. In addition, it has been also reported to be involved in the responses to abiotic stresses including chilling and freezing in plants [21]. Cytokinins (such as tZR) are essentially involved in various plant developmental processes including cell division and enlargement, chloroplast biogenesis, nutrient mobilization and leaf senescence [22], as well as facilitate the responses to delay both stomatal closure and leaf senescence under abiotic stresses [22]. Auxin such as IAA can promote root initiation and also delay plant senescence [23]. Bioactive GAs such as GA_3_ is involved in plant growth and development such as leaf expansion, stem elongation and flowering [22]. Jasmonic acid (JA) is a critical signaling molecule for diverse developmental processes and defense responses in plants [24], and has been shown to induce a response similar to that of ABA for alleviating chilling injury [25]. Salicylic acid (SA) has also been demonstrated to be involved in the responses of plants to a broad range of abiotic stresses, including low temperature [26].

Cold acclimation and the acquisition of freezing tolerance in plants is highly dependent upon the induction of stress-related genes, such as the dehydration-responsive elements or C-repeat binding factor genes (*CBF/DREB1*) as well as the cold-induced stress proteins such as late-embryogenesis abundant (LEA) proteins [7, 27]. Low temperature up-regulated the expression of *CBF/DREB1* which in turn induce the expression of *COLD-REGULATED* (*COR*) genes that to enhance freezing tolerance [28]. The accumulation of LEA functions in direct protection from freezing-induced cellular dehydration and mechanical damage [11].

Zoysiagrass (*Zoysia* spp.) is one of the most widely used warm-season turfgrasses in home lawns, athletic fields and parks because of its good performance to high temperature, water deficit, traffic tolerance and low maintenance [23, 29]. The optimum temperature for the growth and development of zoysiagrass is from 25°C to 30°C. Thus, low temperature stress is one of the essential environmental imiting factors for the geographic distribution and growing season of zoysiagrass in transitional and temperate regions [30]. The native distribution of the recognized species in this genus extends from New Zealand to the island of Hokkaido in Japan, and from French Polynesia through Malaysia west to Mauritius [31]. In eastern China, their natural distribution covers from latitude 19°03′ N to 41°02′ N and from longitude 109°03′ E to124°04′ E [32]. Among all the warm-season turfgrasses, however, zoysiagrass is the most tolerant species in freezing tolerance than other ones, but the cold injuries during the winter season vary widely among zoysiagrass species and genotypes [29]. Our preliminary investigation in a field study showed that zoysiagrass native to high latitude exhibit great freezing tolerance than the ones native to low latitude. The physiological, biochemical and molecular basis for those differences has only partially been explored. The objective of this study was to investigate the cold stress response in zoysiagrass native to diverse latitude at phenotypic, physiological, metabolic and molecular levels under environment-controlled conditions.

## Materials and methods

### Plant materials and growth conditions

Two zoysiagrass *(Zoysia japonica* Steud.) genotypes collected from diverse latitudes (Table 1) were propagated vegetatively in the fields at the Turfgrass Research Farm of Hunan Agricultural University, Changsha, China. Mature zoysiagrass sod plugs were taken from the field plots and transplanted to the pots (18 cm diameter, 20 cm deep) filled with a mixture of top soil and sand (4:1, w/w) in a temperature-controlled greenhouse with the temperature of 30/25°C (day/night) and a photosynthetic active radiation (PAR) at 400 μmol.m^–2^.s^–1^ with 12-h photoperiod. Plants were watered 3 times per week to keep the growth medium at field capacity, and fertilized biweekly with compound fertilizers with the N:P:K = 15:15:10 at a total amount of 57 kg N ha^-1^. Turf was cut 3 times per week at about 4 cm height. Plants were kept in the above mentioned conditions for about two month to allow the full establishment of turf canopies and root systems.

**Table 1:** Origin of the collected zoysiagrass genotypes.

Genotype	Latitude	Longitude	Altitude	Location
Latitude-40	N 40°18.457	E 123°34.602	80 m	Dayingzhi Town, Xiuyan County, Liaoning Province
Latitude-22	N 22°53.435	E 112°16.945	18 m	Yaogu Town, Yunfu City, Guandong Province

### Treatments and experimental design

**Fig 1:**
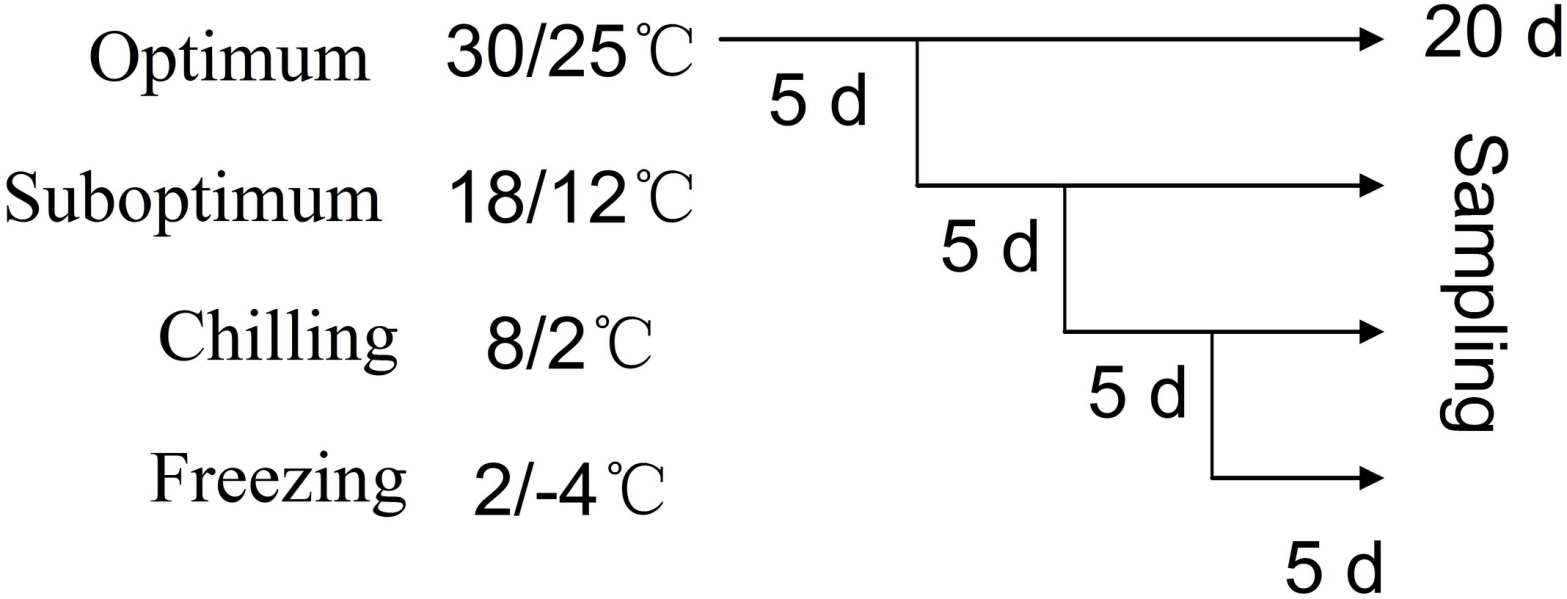
A schematic diagram of the low temperature treatment.

The experiment consisted of four treatments with four replicates in each treatment and a schematic diagram of the low temperature treatment as show in Fig 1: optimum (30/25°C, day/night), suboptimum (18/12°C), chilling (8/2°C) and freezing (2/-4°C). Two months after transplanting, pots were placed in four growth chambers and subjected to temperature treatment with a PAR of 400 μmol·m^–2^·s^–1^ with 12-h photoperiod for a period of 20 d. The low temperature treatment were progressive decreased from 30/25°C, 18/12°C, 8/2°C and 2/-4°C. The irrigation was provided only in need to make sure no moisture stress occurred. No fertilization was provided during 20 d of cold treatment.

### Measurements

#### Turf quality and canopy height

Turf quality was evaluated visually based on the turfgrass color, plant density and uniformity on a 1–9 scale according to Hu et al. (2016) [33]described. Turf canopy height were determined before and after treatment using a ruler according to the method described by Hu et al. (2016) [33].

#### Leaf electrolyte leakage (EL) and LT50

Leaf electrolyte leakage (EL) was determined with about 0.1 g fresh leaf segments collected from each pot and incubated in 15 mL of distilled deionized water on a shaker for 24 h. The initial conductance (Ci) was determined using a conductance meter (YSI-3100; Guangzhou, China). Leaf tissues in the incubation solution were then autoclaved at 120°C for 30 min. The maximum conductance (Cmax) of the incubation solution with killed tissues was determined after the solution cooled to room temperature. Relative EL was calculated as (Ci/Cmax)×100.

The lethal temperature for 50% loss of electrolytes (LT_50_) was performed on the two genotypes after cold acclimation (before starting freezing treatment) to determine their level of freeze tolerance according to Xuan et al. (2013) [34]. Leaves were cut from the plants (10 leaves for each freezing temperature of 0, -2, -6, -10 and -14°C) and maintained in a programmed freezer (Polyscience 9610, Polysciences, Inc., U.S. Corporate Headquarters, Pennsylvania, USA) with a temperature error of ± 0.1°C. The freezing temperature was decreased at a rate of 2°C h^-1^ and then held at each freezing temperature for 1.5 h. The samples were then removed from the freezer and thawed at 2°C overnight. The EL of the leaves collected after freezing was determined by measuring the electrical conductivity of tissues as described above. The LT_50_ values were estimated from a linear regression fitted to the central (linear) part of the sigmoid relationship between the freezing temperature and electrolyte leakage at the five temperatures [34].

#### Leaf chlorophyll content

Leaf chlorophyll content was determined by immersing the fresh leaves (about 0.15 g) in 15 mL of dimethyl sulfoxid and kept in the dark for about 72 h according to Hiscox and Israelstam (1979) [35]. The OD663 and OD645 of supernatants were determined using a spectrophotometer (UV-2600, UNICO (Shanghai) Instruments Co., Ltd., Shanghai, China) after centrifuged at 5000 g for 10 min at room temperature. The concentration of chlorophyll (mg·g^–1^ fresh weight) was calculated using Arnon (1949) [36] equations.

#### Leaf photochemical efficiency and photosynthetic gas exchange

Leaf photochemical efficiency (Fv/Fm) was determined with a chlorophyll fluorometer (OS1-FL, Opti-Sciences, Hudson, NH) on the intact leaves after plants adapted in darkness for 30 min.

Photosynthetic gas exchange measurements (net photosynthetic rate, P_n_; stomatal conductance, g_s_; intercellular CO_2_ concentration, C_i_; transpiration rate, T_r_) were measured with the infrared gas analyzer (Li-6400XT, LICOR, Inc., Lincoln, NE). The measurements environment were set at 400 μmol mol^–1^ CO_2_, 500 μmol s^–1^ flow rate and 500 μmol m^–2^s^–1^ light intensity at 25°C with three subsamples in each pot and totally twelve records in each treatment.

#### Leaf carbohydrates determination

Leaf carbohydrates (glucose, fructose, sucrose, fructan, trehalose, starch) were extracted according to Zhang et al. (2012) [37]. Briefly, 0.1 g of leaf sample was ground in liquid nitrogen and then the tubes were shaken vigorously for 10 min after added 1 mL ethanol (92%, v/v). The extracts were centrifuged at 20,000 × g for 10 min at room temperature. The contents in total soluble carbohydrates were determined using a spectrophotometer (UV-2600, UNICO (Shanghai) Instruments Co., Ltd., Shanghai, China) by mixing 3 mL anthrone reagent (150 mg anthrone and 100 mL of 72% sulfuric acid) with 100 μL supernatant under 625 nm after boiling for 10 min. The remaining supernatant was evaporated to dryness at 40°C in a concentrator, and resolubilized in 300 μL deionized water for extraction of glucose, fructose, sucrose and trehalose. 0.5 mL of deionized water was added and the tubes were heated at 100°C for 10 min. The intermixture was enzymolyzed with 0.1 mL α-amylase (400 U/mL) and 0.1 mL starch transglucosylase (2U/mL) in 0.4 mL acetic buffer (200 mM, pH 5.1) at 55°C for 20 h and centrifuged at 20,000 x g for 10 min. The supernatant was kept boiled in acid environment (0.5 M sulfuric acid) for 15 min. The cooled mixture was neutralized with equal amount of sodium hydroxide (1 M) for the analysis of fructan and starch.

Carbohydrates were analyzed by HPLC (Waters, Massachusetts, USA). Sucrose, fructose, glucose and trehalose were separated on a crest amino column (4.6 × 250 mm, 5 μm) and eluted isocratically at 40°C with buffer [50% (v/v) acetonitrile, 50% (v/v) H_2_O] at a flow rate of 1.0 mL/min. Online detection was performed using a Waters 410 differential refractometer detector, and the data were analyzed by Empower® software. Sucrose, glucose and fructose were used as the standards.

#### Leaf hormone extraction and fractionation for HPLC-MS analysis

Abscisic acid (ABA), trans-zeatin riboside (tZR), indole actic acid (IAA), gibbrelic acid (GA_3_), jasmonic acid (JA) and salicylic acid (SA) were extracted and purified according to the method describe by Dobrev and Kaminek (2002) [38]. The extracted and purified elute was evaporated in a vacuum concentrator at 40°C (MiniVac Beta, LABOGENE, Danmark) and then reconstituted with 2 mL of 1 M formic acid. After washing with 1 M formic acid, ABA and auxins were eluted with 2 mL of methanol, and cytokinins were eluted with 2 mL of 0.35 M ammonia in 60% (v/v) methanol from an Oasis MCX 96-Well Plate as indicated by Dobrev and Kaminek (2002) [38]. Each fraction was evaporated to dryness in a vacuum concentrator and the residues were dissolved in a 500 μl of water/methanol (70/30, v/v) mixture. Prior to injection, dissolved fraction was filtered through 13-mm-diameter Millex filters with a 0.22-μm-pore nylon membrane (Millipore, Bedford, MA, USA) and the samples were transferred to 2-mL HPLC vials.

Hormones analysis were carried out on a high-performance liquid chromatography (HPLC)-mass spectrometry (MS) system (Accela; Thermo Fisher Scientific, San José, CA, USA) according to Ghanem et al. (2008) [39]. For each sample, 10 μL was injected into a Zorbax SB-C18 HPLC column (3.5 μm, 150×2.1 mm, Agilent Technologies) maintained at 35°C and eluted at a flow rate of 200 μL min^-1^. The level of plant hormones tZR, ABA, IAA, GA_3_, SA, JA) in the samples were determined based on retention times and ion products and standards of each compound.

#### Gene expression analysis

Total RNA extraction, cDNA synthesis and qRT-PCRs were performed according to Hu et al. (2016) [33] described. Briefly, total RNA was extracted from fresh tissues using Trizol reagent (Invitrogen, Carlsbad, CA). The first strand cDNA fragments were synthesized from 2 μg of total RNA using oligo(dT)12-18 primer using cDNA synthesis kit (Fermentas, Burlington, Ontario, Canada) after RNA quality and integrity was checked by Nanodrop 2000 and 0.8% agarose gel. Gene-specific primers (Table 2) were designed based on the target gene sequences using Primer 5 software. The qRT-PCRs were performed with ABI7500 in a final volume of 20 μL, with each containing 2 μl of cDNA, 10 μL of 2×SYBR Green qPCR Mix (Takara, Otsu, Shiga, Japan) and 2 μM of the forward and reverse primers. Three independent biological replicates of each sample and two technical replicates of each biological replicate were used for real-time PCR analysis. The thermal cycling conditions were as follows: 40 cycles of 95°C denaturation for 5 s, and 52~55°C annealing and extension for 20 s. To determine relative fold differences for each sample, the CT value for each gene was normalized to the CT value for the reference gene and was calculated relative to a calibrator using the DDCT method as described by Livak and Schmittgen (2001) [40].

**Table 2:** Genes and primers used in this study.

Gene		Primer sequence
*ZjCBF*	F	5'-CGC ATA GCA CTGATC GTC ACC CG-3'
	R	5'-CCT CAC CGC CGT CAT CAT CGT C-3'
*ZjLEA3*	F	5'-TGGGTAGTCAGCCTGGTAGACG-3'
	R	5'-TGGGTAGTCAGCCTGGTAGACG-3'
*ZjDREB1*	F	5'-TTGGAGGCTGCTCATGCATA-3'
	R	5'-TTCAACGCATGCACCTCAGT-3'
*Actin*	F	5'-TGT GCT CAG CGG TGG TTC AA-3'
	R	5'-TGC TGG GCC AGA CTC GTC AT-3'

### Statistical analysis

All data were subjected to two-way analysis of variance using SAS software (v. 9.3 for Window; SAS Institute, Cary, NC, USA, 2010). The treatment means were separated using Ducan’s multiple range test at the *P* < 0.05 probability level.

## Results

### Freezing tolerance (LT50 and EL)

**Fig 2:**
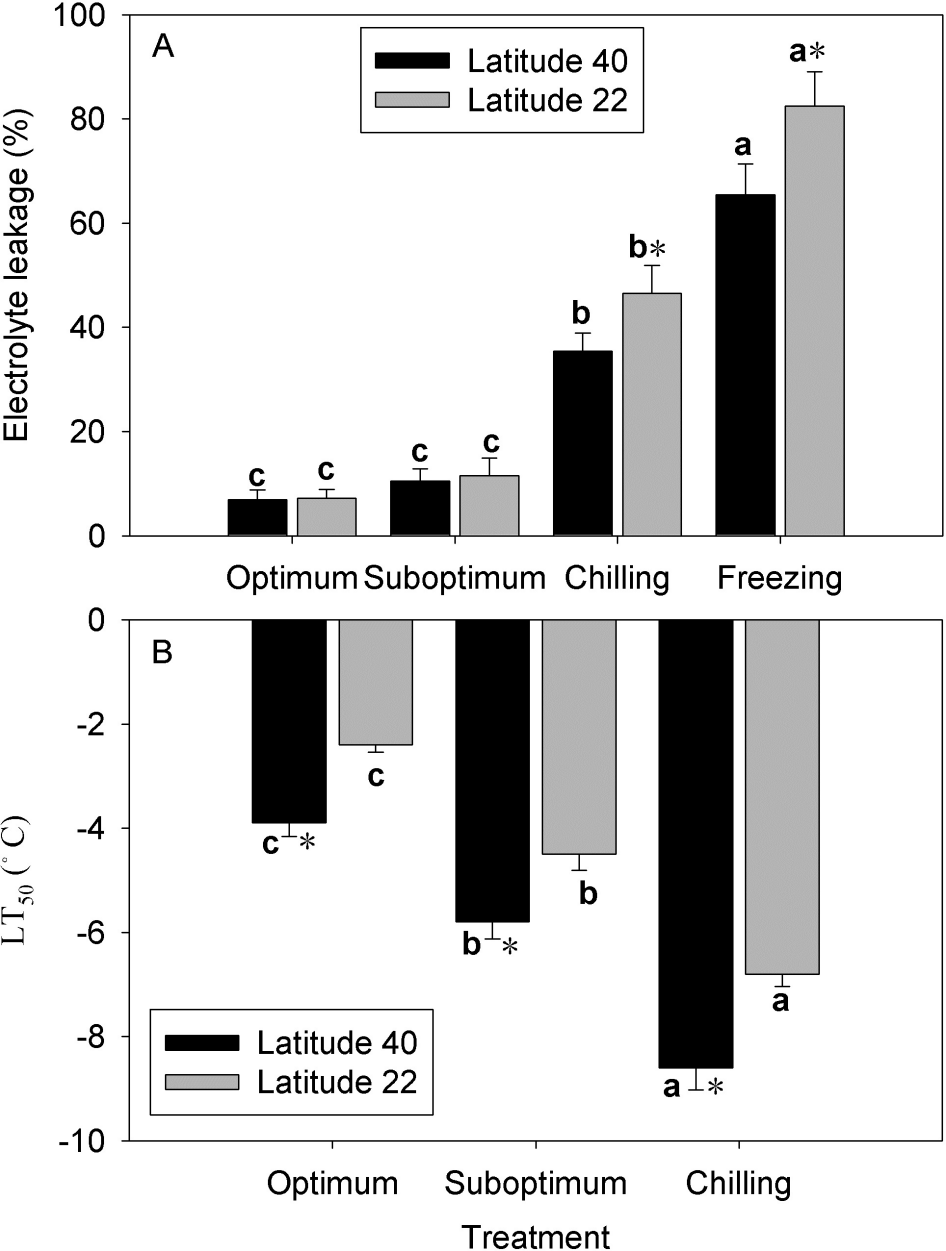
Effect of low temperature on the leaf electrolyte leakage (EL, A) and lethal temperature for 50% loss of electrolytes (LT_50_, B) in two zoysiagrass genotypes native to diverse latitude. Vertical bars on the top indicate standard deviation, and bars with the same letter indicate no significant difference at *P* < 0.05 for the comparison of differential temperature treatment at a given genotype, and * on the top indicate significant difference at *P* < 0.05 between two genotypes at a given temperature treatment.

Suboptimum temperature (18/12°C, day/night) has no significant effect on the leaf EL for two zoysiagrass genotypes (Fig 2A). However, chilling and freezing temperature significantly increased leaf EL for both genotypes, with more increasing in Latitude-22 than in Latitude-40, which had 11.1% and 17.1% higher in Latitude-22 under chilling and freezing temperature, respectively.

Under optimum temperature conditions, the freezing tolerance (LT_50_) of Latitude-40 and Latitude-22 were -3.9 and -2.4°C (Fig 2B). After exposure to cold acclimating conditions, the leaves freezing tolerance (LT_50_) of the two zoysiagrass genotypes decreased from -2.4 to -4.5°C and -6.8°C for Latitude-22, and decreased from -3.9 to 5.8°C and -8.6°C for Latitude-40 under suboptimum and chilling temperature, respectively.

### Turf quality and canopy height

**Fig 3:**
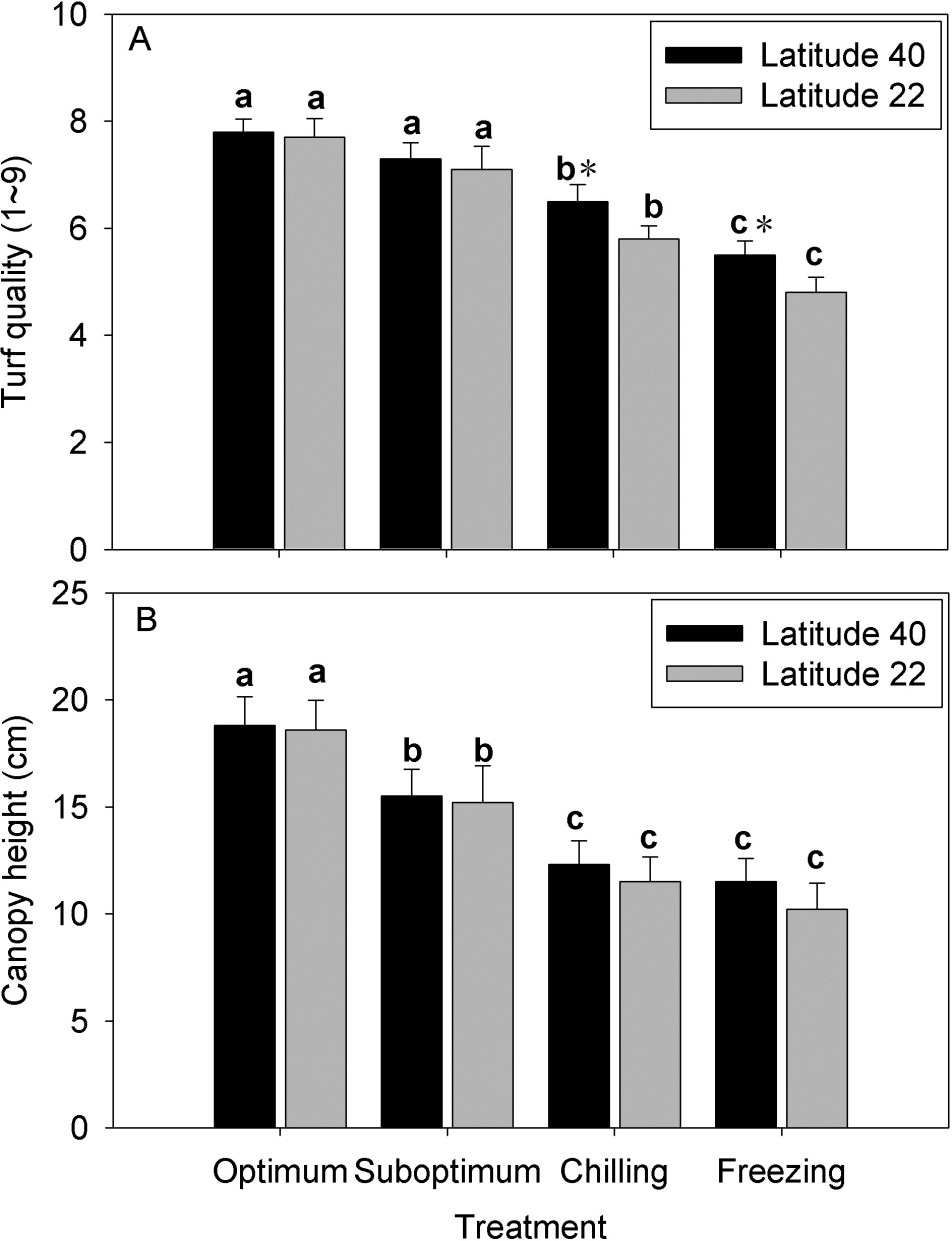
Effect of low temperature on the turf quality (A) and canopy height (B) in two zoysiagrass genotypes native to diverse latitude. Vertical bars on the top indicate standard deviation, and bars with the same letter indicate no significant difference at *P* < 0.05 for the comparison of differential temperature treatment at a given genotype, and * on the top indicate significant difference at *P* < 0.05 between two genotypes at a given temperature treatment.

There was no significant reduction in turf quality for both genotypes under suboptimum temperature (Fig 3A). Chilling and freezing temperature caused significantly decrease in turf quality for both genotypes, to a great extent for Latitude-22. Decreasing temperature reduced plant growth as indicated by the canopy height, but no significant difference was observed between the two genotypes at each temperature (Fig 3B).

### Leaf Chl content and photochemical efficiency (Fv/Fm)

**Fig 4:**
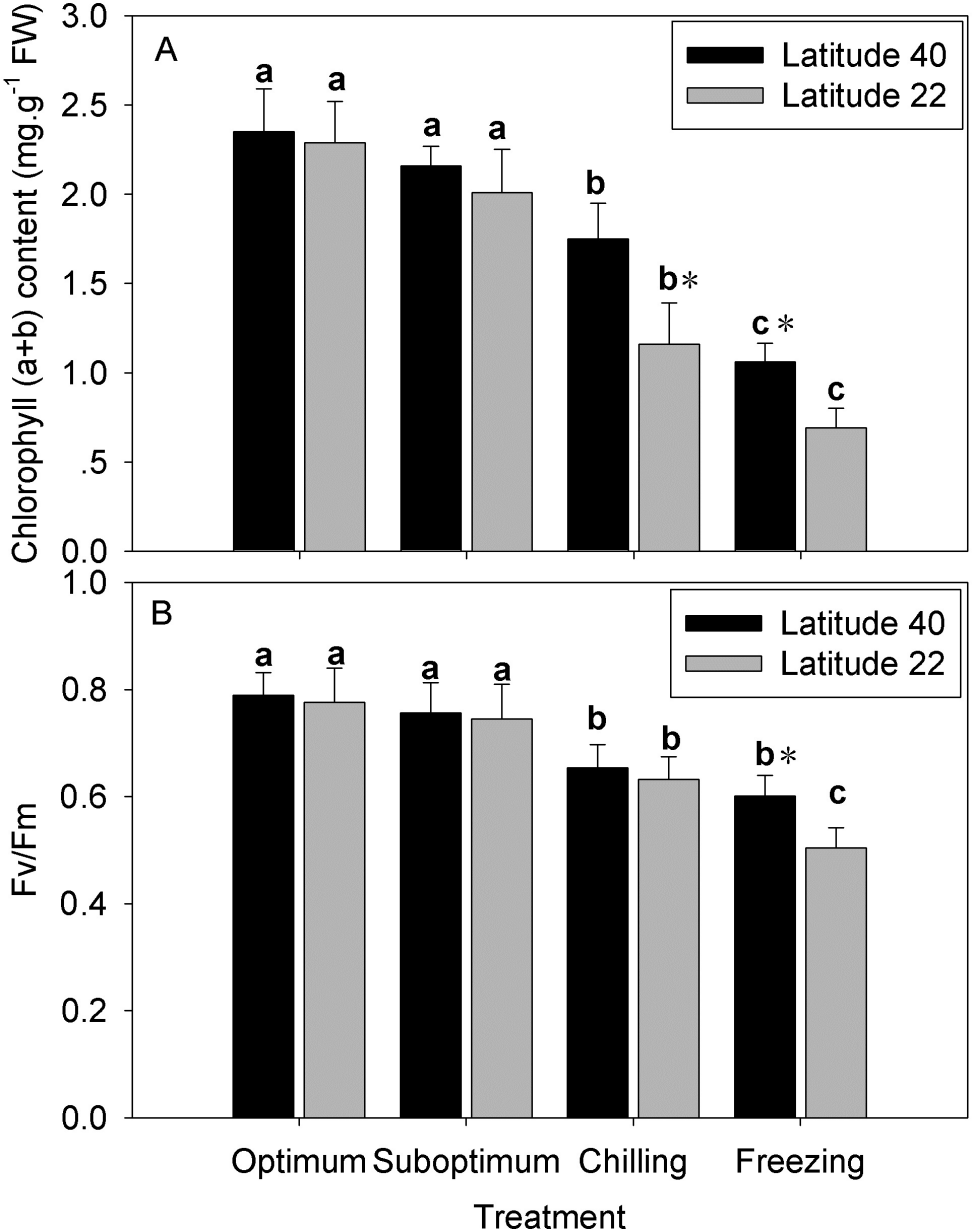
Effect of low temperature on the total chlorophyll content in two zoysiagrass genotypes native to diverse latitude. Vertical bars on the top indicate standard deviation, and bars with the same letter indicate no significant difference at *P* < 0.05 for the comparison of differential temperature treatment at a given genotype, and * on the top indicate significant difference at *P* < 0.05 between two genotypes at a given temperature treatment.

No significant reduction in chlorophyll content (Chl, Fig 4A) and Fv/Fm (Fig 4B) for both genotypes under suboptimum temperature. Chilling and freezing temperature caused significantly decrease in Chl and Fv/Fm for both genotypes, to a great extent for Latitude-22. Chlorophyll content decreased to 92%, 74% and 45% of the optimum temperature levels for Latitude-40, and decreased to 88%, 51% and 30% of the optimum temperature levels for Latitude-22 at suboptimum, chilling and freezing temperature, respectively (Fig 4A). Fv/Fm decreased to 83% and 76% of the optimum temperature levels for Latitude-40, and decreased to 81% and 67% of the optimum temperature levels for Latitude-22 at chilling and freezing temperature, respectively (Fig 4B).

### Leaf photosynthetic gas exchange

**Fig 5:**
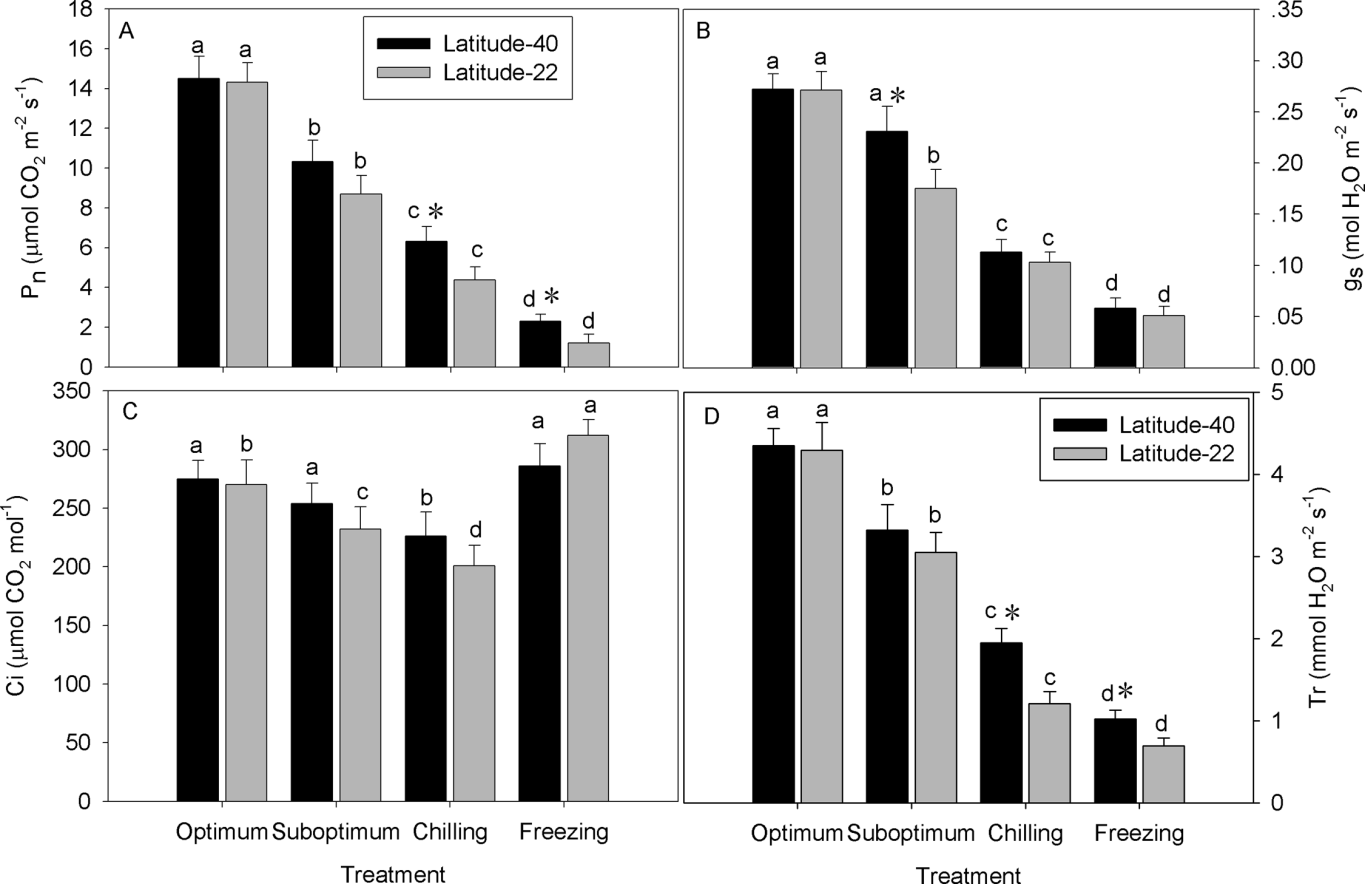
Effect of low temperature on the total chlorophyll content in two zoysiagrass genotypes native to diverse latitude. Vertical bars on the top indicate standard deviation, and bars with the same letter indicate no significant difference at *P* < 0.05 for the comparison of differential temperature treatment at a given genotype, and * on the top indicate significant difference at *P* < 0.05 between two genotypes at a given temperature treatment.

Leaf P_n_ declined with the temperature decreasing in both genotypes, to a great extend in Latitude-22 (Fig 5A). Leaf P_n_ decreased to 71%, 43% and 16% of the optimum temperature levels for Latitide-40, and decreased up to 60%, 30% and 8% of the optimum temperature levels for Latitide-22 at suboptimum, chilling and freezing temperature, respectively (Fig 5A). Suboptimum temperature decreased g_s_ in Latitude-22 but not in Latitude-40, whereas chilling and freezing temperature decreased leaf g_s_ in both genotypes (Fig 5B). Suboptimum temperature decreased leaf C_i_ in Latitude-22 but not in Latitude-40, whereas chilling decreased leaf g_s_ in both genotypes. However, leaf g_s_ increased to the optimum temperature level in Latitude-40, even higher than the optimum temperature level in Latitude-22, and no significant difference was observed between the two genotypes (Fig 5C). Leaf T_r_ declined with the reduction of temperature in both genotypes, with more rapid decrease in Latitude-22 than Latitude-40 (Fig 5D). Leaf T_r_ decreased to 76%, 45% and 23% of the optimum temperature level for Latitude-40, while decreased up to 71, 28 and 16% of optimum temperature level for Latitude-40 under suboptimum, chilling and freezing temperature (Fig 5D).

### Leaf carbohydrates content

Leaf carbohydrates content generally increased with the reduction of temperature in two genotypes (Table 3). Sucrose and fructose content remarkably increased in both genotypes under low temperature, with more rapid increase in Latitude-40 than in Latitude-22. Sucrose levels increased by 3.1-, 7.1- and 7.9-fold in Latitude-40, and increased by only 1.8-, 3.8- and 5.3-fold in Latitude-22 at suboptimum, chilling and freezing temperature, respectively. Fructose content increased by to 1.6-, 3.3- and 4.5-fold for Latitude-40, and increased by 1.1-, 1.9- and 2.8-fold for Latitude-22 at suboptimum, chilling and freezing temperature, respectively.

**Table 3:** Effect of low temperature on carbohydrate content in leaves of two zoysiagrass genotypes.

Treatments	Genotypes	Carbohydrates content (mg/g DW)						
		Sucrose	Glucose	Fructose	Trehalose	Fructan	Starch	TSS
Optimum	Latitude 40	8.3±0.56 a	23.5±0.34 a	7.6±0.86 a	3.5±0.43 a	45.3±3.75 a	72.5±5.64 a	52.3±4.31 a
	Latitude 22	8.5±0.76 a	22.4±0.29 a	7.9±0.49 a	2.9±0.38 a	42.3±3.02 a	73.7±6.35 a	50.5±3.45 a
Suboptimum	Latitude 40	25.6±3.21 a	28.5±3.21 a	12.5±1.58 a	16.5±1.97 a	68.5±5.34 a	93.5±6.34 a	95.1±5.64 a
	Latitude 22	15.6±2.14 b	25.3±2.12 a	8.6±1.34 b	6.5±1.12 b	47.6±3.78 b	79.3±5.31 b	68.3±4.31 b
Chilling	Latitude 40	58.6±5.31 a	38.6±2.98 a	25.3±1.78 a	25.7±1.71 a	94.5±6.78 a	113.4±8.64 a	153.1±8.64 a
	Latitude 22	32.5±4.12 b	28.5±3.45 b	15.4±1.13 b	12.5±1.35 b	68.3±4.58 b	92.1±9.31 b	98.3±7.61 b
Freezing	Latitude 40	65.4±5.34 a	42.3±3.42 a	34.1±2.12 a	52.1±3.97 a	110.5±7.34 a	62.5±4.36 b	210.2±9.31 a
	Latitude 22	45.3±3.98 b	32.1±2.97 b	22.1±1.97 b	21.2±4.31 b	88.4±4.31 b	93.5±6.84 a	143.5±9.84 b

Note: Means followed by the same lower-case letters in a column within a temperature treatment indicated no significant difference between two genotypes at *P*<0.05.

There were no significant difference in glucose content between two genotypes under optimum and suboptimum temperature, and the glucose levels were 26 and 24% higher in Latitude-40 than in Latitude-22 under chilling and freezing temperature respectively (Table 3).

Trehalose, fructan and total soluble sugar (TSS) levels considerably enhanced as the temperature decline, to a great extend for Latitude-40 (Table 3). Trehalose content increased by 2.2-, 4.3- and 7.3-fold in Latitude-22, whereas increased by up to 4.7-, 7.3- and 14.9-fold in Latitude-40 at suboptimum, chilling and freezing temperature, respectively. Starch content significantly increased at suboptimum and chilling temperature and then decline at freezing temperature for Latitude-40. For Latitude-22, however, starch levels increased at chilling and freezing temperature, and no significant change at suboptimum temperature.

### Leaf hormone content

Leaf ABA, SA and JA levels generally increased with the decrease of temperature in both genotypes, with more rapid increase in Latitude-40, whereas IAA, tZR and GA_3_ levels remarkably declined with the reduction of temperature in two genotypes, to a great extend in Latitude-22 (Table 4). Leaf ABA content increased by 1.5-, 2.9- and 3.1-fold for Latitude-40, and increased by 1.2-, 2.3- and 2.4-fold for Latitude-22 at suboptimum, chilling and freezing temperature. There were no significant difference in leaf IAA and tZR levels between two genotypes under suboptimum temperature, but maintained a relatively higher levels in Latitude-40 than in Latitude-22 under chilling and freezing temperature (Table 4). Leaf GA_3_ content significantly declined under suboptimum, chilling and freezing temperature for both genotypes, and no significant difference between the two genotypes.

**Table 4:** Effect of low temperature on hormones content in leaves of two zoysiagrass genotypes.

Treatments	Genotypes	Hormones content (ng/g FW)					
		ABA	IAA	tZR	GA_3_	SA	JA
Optimum	Latitude 40	23.7±1.94 a	76.5±5.34 a	45.6±3.45 a	8.3±0.65 a	10.3±1.24 a	9.3±1.10 a
	Latitude 22	24.7±2.02 a	72.3±4.34 a	47.3±2.98 b	7.9±0.59 a	9.8±1.17 a	8.8±0.97 a
Suboptimum	Latitude 40	35.5±2.97 a	62.3±4.34 a	39.3±2.64 a	6.5±0.49 a	11.5±1.09 a	14.3±1.54 a
	Latitude 22	30.5±2.01 b	58.5±3.97 a	36.7±3.01 a	6.3±0.67 a	12.5±1.35 a	8.9±0.94 b
Chilling	Latitude 40	68.6±4.35 a	50.4±3.94 a	30.4±2.81 a	4.3±0.52 a	18.4±1.12 a	19.3±2.14 a
	Latitude 22	56.4±3.97 b	35.9±2.78 b	21.5±1.97 b	5.0±0.39 a	14.5±1.71 b	12.3±1.57 b
Freezing	Latitude 40	72.3±5.34 a	34.2±2.04 a	19.4±1.12 b	1.9±0.15 a	23.8±1.47 a	21.3±2.23 a
	Latitude 22	58.3±4.64 b	21.4±3.12 b	10.5±1.08 b	1.6±0.25 a	18.6±1.68 b	15.8±1.84 b

Note: Means followed by the same lower-case letters in a column within a temperature treatment indicated no significant difference between two genotypes at *P*<0.05.

Leaf SA levels remarkably increased with the temperature decline, with more rapid increase in Latitude-40 than in Latitude-22 (Table 4). Leaf SA levels increased by 1.3-, 1.5- and 1.9- fold for Latitude-22, whereas increased by up to 1.1-, 1.8- and 2.3-fold for Latitude-40 at suboptimum, chilling and freezing temperature, respectively. Leaf JA content increased by 1.5-, 2.1- and 2.3-fold in Latitude-40, and increased by only 1.0-, 1.4- and 1.8-fold in Latitude-22 at suboptimum, chilling and freezing temperature (Table 4).

### Cold stress related gene expression

**Fig 6:**
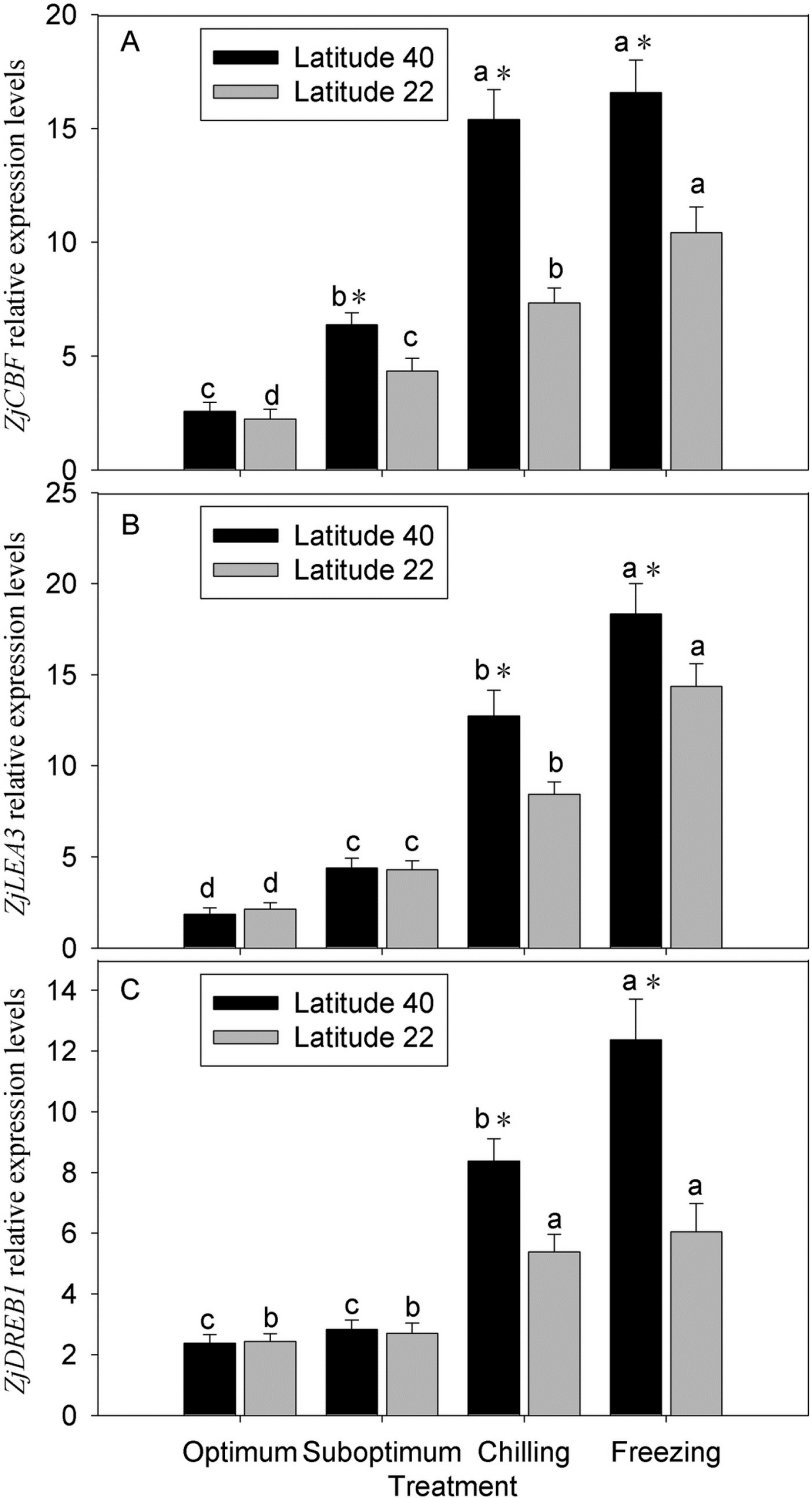
Effects of low temperature on the cold induced gene expressions in leaves of two zoysiagrass genotypes native to diverse latitude. Vertical bars on the top indicate standard deviation, and bars with the same letter indicate no significant difference at *P* < 0.05 for the comparison of differential temperature treatment at a given genotype, and * on the top indicate significant difference at *P* < 0.05 between two genotypes at a given temperature treatment.

Temperature reduction induced up-regulation of *ZjCBF* and *ZjLEA3* expressions in leaves of two zoysiagrass genotypes, with more increasing in Latitude-40 than in Latitude-22 (Fig 6A and 6B). The expression levels of *ZjCBF* gene increased by 2.8-, 5.9- and 6.4-fold for Latitude-40, whereas increased by only 1.9-, 3.3- and 4.7-fold for Latitude-22 at suboptimum, chilling and freezing temperature, respectively (Fig 6A). The *ZjLEA3* gene expression levels increased by 2.0-, 3.9- and 6.7-fold in Latitude-22, while increased by up to 2.4-, 6.9- and 9.9-fold in Latitude-40 (Fig 6B). The *ZjDREB1* gene expression level was not induced by suboptimum temperature, and considerably induced by chilling and freezing temperature in leaves of two zoysiagrass genotypes (Fig 6C). The *ZjDREB1* gene expression levels increased by 2.2- and 2.5-fold in Latitude-22, whereas increased by up to 3.5- and 5.2-fold in Latitude-40 (Fig 6C).

## Discussion

Temperature may be the main environmental factor determining the natural latitudinal and altitudinal distribution of plants. The native distribution of zoysiagrass in China extends diverse latitude covers from 19°03′ N to 41°02′ N [32], which showed greater freezing tolerance than other warm-season turfgrasses and vary cold tolerance among zoysiagrass species and genotypes [29]. Two representative genotypes were chosen for this study based on our preliminary investigation in a field study, which demonstrated that the genotype native to higher latitude exhibit higher freezing tolerance than the ones native to warmer latitude. In this study, we further confirmed the result in a controlled environment that genotypes native to higher latitude showed higher freezing tolerance as indicated by the EL and LT_50_ under both cold-acclimated and non-acclimated conditions. It has been suggested that the enhanced freeze tolerance in plants partially attribute to the cold acclimation [31]. The leaf LT_50_ was from -3.9°C to -8.6°C for the higher latitude genotype and from -2.4°C to -6.8°C for the lower latitude genotype under optimum to chilling temperature, suggesting that cold acclimation improved freeze tolerance for zoysiagrass regardless of their latitude origins.

Low temperature caused reduction in photosystem performance and photosynthetic apparatus activities have been associated with photo-inhibition arised from the reduced capacity for energy utilization [3]. In our study, leaf P_n_, g_s_ and T_r_ remarkably declined with the reduction of temperature, along with a great decrease in plant canopy height, suggesting that the cessation of growth resulting from cold stress reduces the capacity for energy utilization, causing feedback inhibition of photosynthesis during the initial phase of chilling stress [41]. Differences in the capacity to minimize photo-inhibition of photosynthesis during low temperature have been implied to contribute to the intra- and inter-specific differences in freezing tolerance [10]. In this study, genotype Latitude-40 exhibited higher photosynthesis under low temperature compared with Latitude-22, suggesting that genotypes native to higher latitude may have a higher capacity to minimize the photo-inhibition of photosynthesis that leading to the improved freezing tolerance.

A decrease in Fv/Fm indicated photosynthesis reduction or photo-inhibition under environmental stress [42]. The results of this study showed that Fv/Fm did not decline under suboptimum temperature until chilling and freezing temperature. These results indicated that photochemical efficiency in zoysiagrass was relatively less sensitive to temperature decline than the g_s_ and C_i_. Thus, the early inhibition of photosynthesis by low temperature in zoysiagrass could not be associated with the damage of PSII at this stage. A similar results in Fv/Fm was also observed in cool-season turfgrass creeping bentgrass and annual bluegrass subjected to chilling stress or during cold acclimation [43].

Nonstructural carbohydrates (starch, hexose, sucrose) are essentially involved in the recovery from dormancy, re-growth and recuperative capacity from abiotic stress, particular under low temperature [44]. Changes in the accumulation of water soluble sugars (WSC) during low temperature have been reported in many warm- and cool-season turfgrass [12–15, 45]. Soluble carbohydrates have been associated with freeze tolerance and could leading to inter- and intra-specific differences in plant freezing tolerance in various warm-season turfgrasses [12–13, 45]. In our study, total WSC (TSS) significantly increased in leaves of both genotypes under low temperature. The genotype Latitude-40 exhibited significantly higher TSS compared to Latitude-22. This result suggests that higher accumulations of TSS in leaves of cold tolerant genotype may account for the improved membrane stability at low temperatures and contributed to the better freezing tolerance.

Sucrose content increased from optimum to freezing temperature for both genotypes. Previous works on cool- and warm-season turfgrasses also showed an increase in sucrose content under low temperature [13, 45]. However, there have been various reports on the associations between the accumulation of sucrose and freezing tolerance in turfgrass species. There were high correlations between surose content and freezing tolerance in warm-season grass [12, 14, 46], whereas no correlations were found between sucrose levels and freezing tolerance in cool-season annual bluegrass (*Poa annua*) [13]. In this study, the genotype native to higher latitude (Latitude-40) exhibited higher sucrose accumulation along with a lower LT_50_ than the ones native to lower latitude (Latitude-22) with the temperature decline, suggesting a positively correlation between the sucrose accumulation and the freezing tolerance in zoysiagrass.

An enhancement in soluble carbohydrates levels during cold acclimation generally along with a reduction in starch levels [9]; however, studies on starch content in stolons and rhizomes of warm-season grasses vary during cold-acclimation. Starch concentration in rhizomes and stolons of bermudagrass and centipedegrass decreased during cold acclimation [14], whereas the starch concentration of carpetgrass (*Axonopus affinis*) and zoysiagrass has been reported to increase during cold acclimation [47–48]. The results of this study are in agreement with the reports that starch content significant increased in zoysiagrass under suboptimum and chilling temperature [47–48]. However, starch content decreased for the genotype native higher latitude and increased for the lower latitude genotype under freezing temperature. These results suggested that cold-acclimated zoysiagrass contain less starch after cold acclimation potentially contributed to carbohydrate accumulation in response to cold leading to more freeze tolerance [17, 49].

Soluble sugars trehalose and fructans are documented to protect plants from freeze-induced dehydration and reduce ice formation by increasing the intracellular solute concentration [18]. In the current study, trehalose and fructan increased with the temperature decline in two genotypes, with higher content in the genotype native to higher latitude along with a lower LT_50_, suggesting those two soluble sugars are important factors in the cold acclimation process and involved in the freezing tolerance in zoysiagrass, which has also been implicated in perennial ryegrass that increased fructan synthesis was positively associated with improved freezing tolerance [50].

ABA has been considered as signaling molecules for inducing plant antioxidant defense systems against abiotic stresses [51]. Cytokinins are reported to promote shoot initiation, lateral bud growth, leaf expansion, nutrient mobilization, chloroplast differentiation, activation of shoot meristems and delay senescence [52]. The results of this study showed that low temperature lead to an increase in ABA and decline in t-ZR content in leaves of zoysiagrass. This is consistent with previous studies in warm-season turfgrass species [23, 53]. It is reported that ABA plays a crucial role in promoting plant tolerance to cold and exogenous application of ABA promotes freezing tolerance in plants [54]. The higher ABA content were observed in the genotype native to higher latitude under low temperature, suggesting that ABA accumulation during cold acclimation is associated with freezing tolerance in zoysiagrass [54].

Endogenous GA content has been reported to declined with the reduction of temperature, which is correlated with the shoot growth inhibition in carrot (*Daucus carota*) [55] and sunflower (*Helianthus annuus*) [28]. In our study, GA_3_ levels significantly reduced in both zoysiagrass genotype. Concomitantly, the cold-stressed plants exhibited lower canopy height and no difference were observed between the two zoysiagrass genotypes. These results suggested that the growth reduction under low temperature may partially attribute to the lowered GA levels in zoysiagrass. It was reported that cold acclimation involves a decrease in endogenous IAA levels in Arabidopsis [56]. However, an increased endogenous IAA concentration was observed in the seedlings of in rice (*Oryza sativa*) seedlings under low temperature stress [57]. In wheat, a significant increase in IAA concentrations was found in crown tissues but not in leaves [58]. In addition, whereas IAA levels were not affected by cold stress in spring wheat and significant increases occurred in winter wheat after cold exposure [59]. In our study, IAA content decreased in leaves of zoysiagrass under low temperature, suggesting that cold stress appears to affect IAA levels with differences depending on plant species, developmental context and organs.

Salicylic acid (SA) has also been reported to be associated with the low temperature stress tolerance in plants [60]. Salicylic acid pretreatment obviously ameliorated the damages of cold stress in several plant species such as banana (*Musa acuminate*) [61], corn (*Zea mays*), cucumber (*Cucumis sativus*) and rice [62] and tomato (*Lycopersicon esculentum*) plants [63]. In our study, subjected to low temperatures increased the endogenous SA levels in zoysiagrass, with higher levels in the genotype native to higher latitude. This result suggested that SA is involved in the cold stress tolerance in zoysiagrass, which may partially attribute to the SA induced CBFs following cold treatment and promoted freezing tolerance, because jasmonate function is a critical upstream signal in ICE1-CBFs pathway to positively regulate low temperature tolerance [25, 64]. In addition to GAs, SA is another hormone that appears to contribute to the low temperature-induced growth retardation of plants. Cold-induced increases in SA levels were reported for both chilling-sensitive and freezing-tolerant plant species [58]. In this study, great growth reduction was observed along with a significant decline in GA_3_ levels but a slight increase in SA levels under suboptimum temperature, whereas a great increase in SA content at chilling to freezing temperature when GA_3_ reduced to a very low levels. These results suggested that SA may act to regulate plant growth inhibition in the later stages of cold treatment or under the severe cold stress, when the role of GA becomes less pronounced.

Stress tolerance in plants is highly dependent upon the induction of specific stress related signaling pathway and genes [64]. The best understood cold signaling pathway is mediated by ICE1/CBF/COR transcriptional cascade. In this sense, the C-repeat (CRT)-binding factors (*CBFs*)/dehydration-responsive elements (*DREBs*) are induced by cold, leading to the induction of cold responsive (*COR*) gene expression associated with freezing tolerance [8]. The current study measured changes in the expression of three cold responsive genes including the *CBF*, *DREB1* and *LEA3*, and those genes significantly up-regulated with the decreasing of temperature in zoysiagrass, whereas the expression levels was higher in the genotype native to higher latitude along with a lower LT_50_. These results suggested that a higher expression of these TFs and genes in zoysiagrass were associated with an increased tolerance to chilling and freezing temperatures, which in agreement with previous studies in Arabidopsis [65], Citrus species [66].

Key components of the ABA and JA signaling pathways have been shown to modulate *CBFs* under low temperature [67]. Moreover, certain *CBF* genes are induced by exogenous application of ABA, contributing to sustain cold tolerance [68]. JA has been shown to induce a response similar to that of ABA for alleviating chilling and freezing injury in plants [25]. The results of this study indicated that low temperature induced up-regulation of *ZjCBF*, *ZjDREB1* and *ZjLEA* genes in leaves of zoysiagrass with the concomitant increase in ABA and JA content, suggesting ABA and JA contribute to the cold tolerance in zoysiagrass served as positive regulators during the signaling pathways under low temperature.

## Conclusions

Zoysiagrass native to higher latitude exhibited higher freezing tolerance, and the higher freezing tolerance may attribute to the higher carbohydrates content serving as energy reserves and stress protectants that stabilize cellular membranes. The higher levels of phytohormones may serve signals in regulating plant growth, development and adaptation to low temperature as well as involved in the signaling pathways that induced the up-regulated *ZjCBF*, *ZjLEA3* and *ZjDREB*1 expressions thus result in a higher cold tolerance.
